# Supplementary material for: In silico detection of SARS-CoV-2 specific B-cell epitopes and validation in ELISA for serological diagnosis of COVID-19
Source: Sci Rep. 2021 Feb 22;11:4290. doi: 10.1038/s41598-021-83730-y (PMC7900118; doi:10.1038/s41598-021-83730-y)
Supplement: Supplementary file 1 — Supplementary Information 1. [file 41598_2021_83730_MOESM1_ESM.docx]

In silico detection of SARS-CoV-2 specific B-cell epitopes and validation in ELISA for serological diagnosis of COVID-19

Isabelle Q. Phan^1,2^, Sandhya Subramanian^1,2^, David Kim^1,3,4,5^, Michael Murphy^3,4^, Deleah Pettie^3,4^, Lauren Carter^3,4^, Ivan Anishchenko^1,3,4^, Lynn K. Barrett^1,6^, Justin Craig^1,6^, Logan Tillery^1,6^, Roger Shek^1,6^, Whitney E. Harrington^2,12^, David M. Koelle^6,8,9,13,16^, Anna Wald^7,8,9,10^, David Veesler^3^, Neil King^3,4^, Jim Boonyaratanakornkit^7,8^, Nina Isoherranen^11^, Alexander L. Greninger^9^, Keith R. Jerome^9^, Helen Chu^6^, Bart Staker^1,2^, Lance Stewart^1,3,4^, Peter J. Myler^1,2,14^, Wesley C. Van Voorhis^1,5,15,16*^

^1^ Seattle Structural Genomics Center for Infectious Disease (SSGCID), Seattle, Washington, USA.

^2^ Center for Global Infectious Disease Research, Seattle Children’s Research Institute, Seattle, Washington, USA.

^3^ Department of Biochemistry, University of Washington, Seattle, Washington, USA.

^4^ Institute for Protein Design (IPD), University of Washington, Seattle, Washington, USA.

^5^ Howard Hughes Medical Institute, University of Washington, Seattle, Washington, USA.

^6^ Center for Emerging and Re-emerging Infectious Diseases (CERID), Division of Allergy and Infectious Diseases, Department of Medicine, University of Washington, Seattle, Washington, USA.

^7^ Division of Allergy and Infectious Diseases, Department of Medicine, University of Washington, Seattle, Washington, USA.

^8^ Vaccine and Infectious Diseases Division, Fred Hutchinson Cancer Research Center, Seattle, Washington, USA.

^9^ Department of Laboratory Medicine, University of Washington, Seattle, Washington, USA.

^10^ Department of Epidemiology, University of Washington, Seattle, Washington, USA.

^11^ Department of Pharmaceutics, University of Washington, Seattle, Washington, USA

^12^ Department of Pediatrics, University of Washington, Seattle, Washington, USA.

^13^ Benaroya Research Institute, Seattle, Washington, USA.

^14^ Department of Medical Education and Biomedical Informatics & Department of Global Health, University of Washington, Seattle, Washington, USA.

^15^ Department of Microbiology, University of Washington, Seattle, Washington, USA.

^16^ Department of Global Health, University of Washington, Seattle, Washington, USA.

*Correspondence to WVanVoorhis@medicine.washington.edu

# Supplemental information

**Figure S1**. Summary plots for each of the 27 SARS-CoV-2 proteins analyzed in this study. The x-axis represents the amino acid positions in the protein sequence. From top to bottom: **A**) BepiPred2 linear epitope predictions are shown as green bars above the black line representing the 80% specificity threshold (0.55), predictions below the threshold are greyed out; **B**) Epitope conservation vs endemic HCoVs as blue bars spanning the length of the trimmed epitope, with the height representing the degree of conservation (we consider values > 60% as likely non-specific), grey bars correspond to continuous, conformational epitopes with a linear prediction score below the threshold; **C**) DiscoTope2 conformational epitope predictions as black bars above the 80% specificity threshold (-2.5 scaled to zero), predictions below the threshold are greyed out; **D**) Shannon Entropy representing residue variability as pink bars; **E**) Variant counts as purple bars representing the number of residue types found at that position, that differ from the reference sequence.


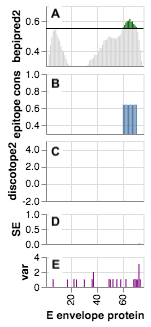

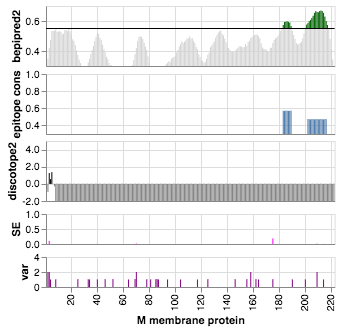


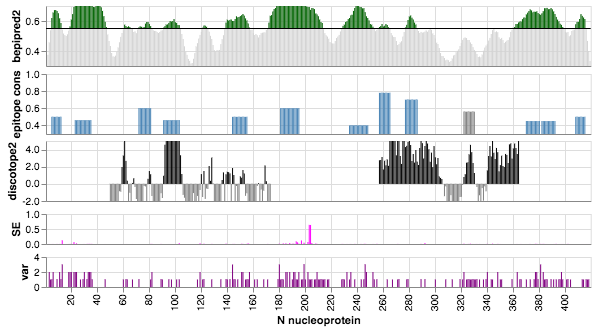

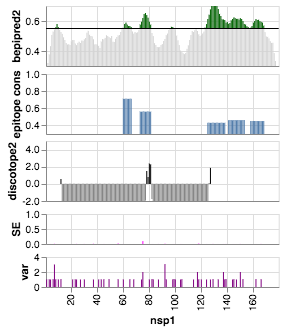

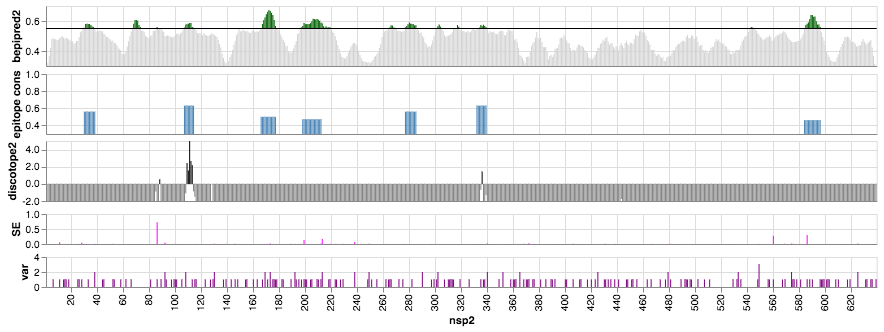

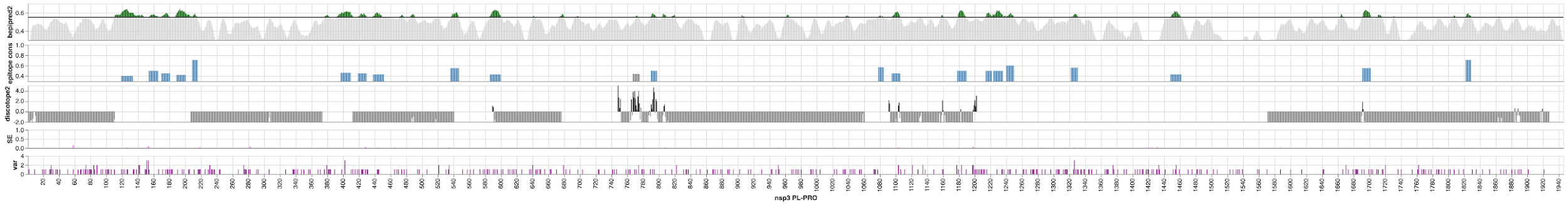

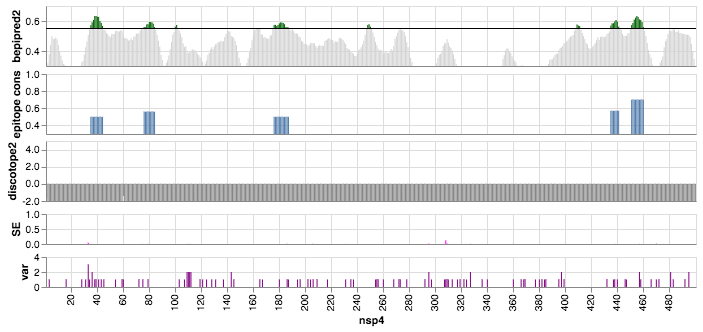

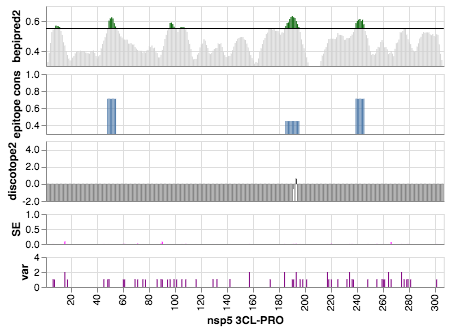

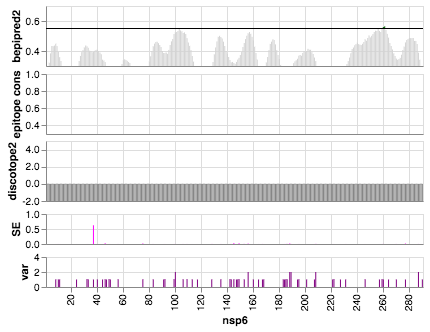

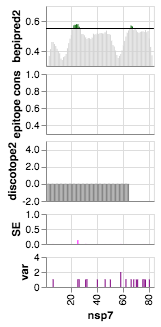

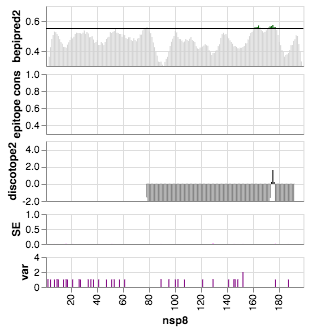

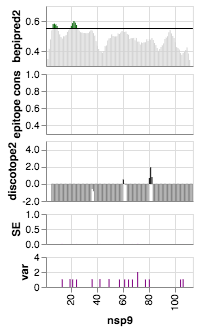

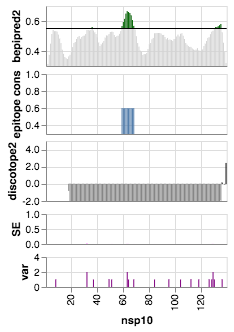

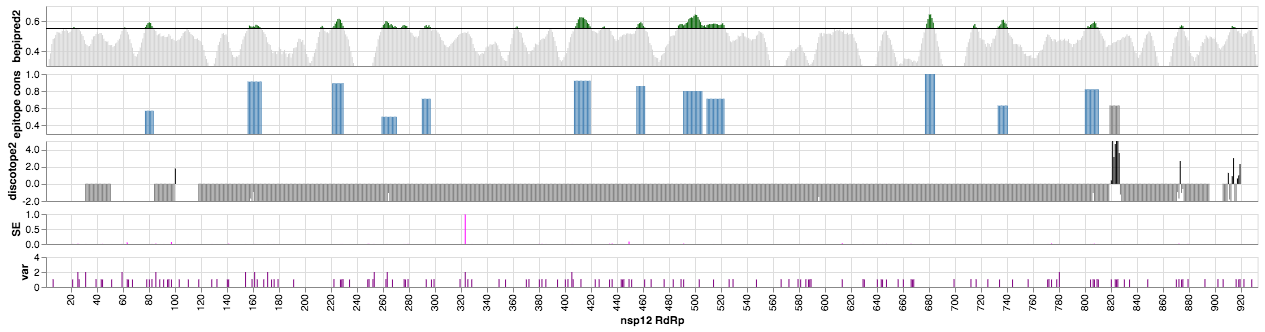

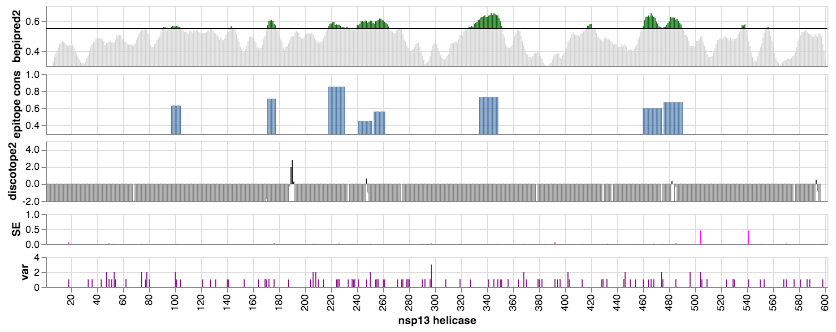

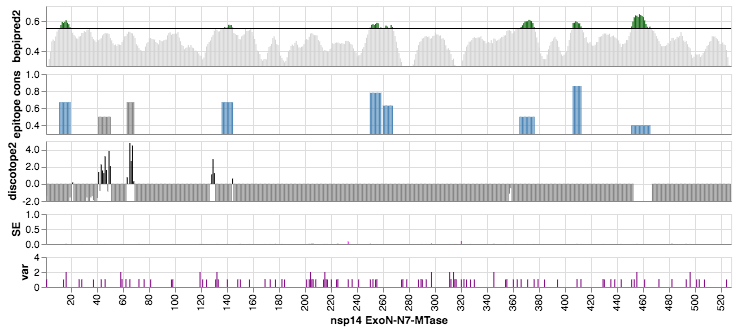

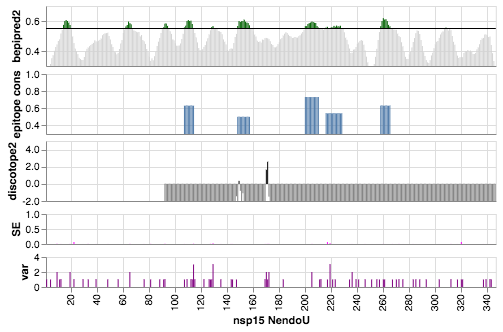

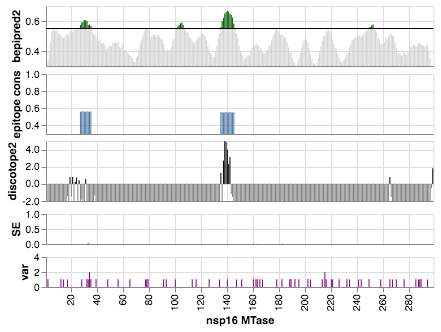

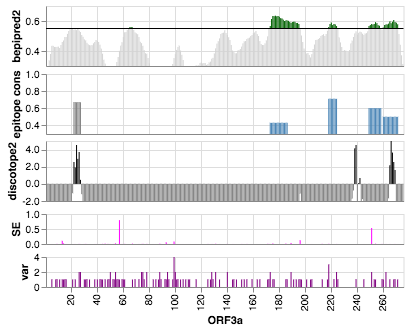

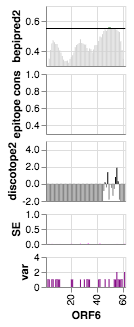

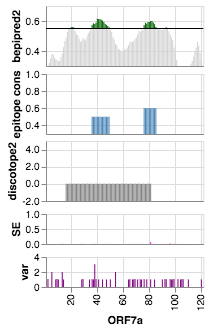

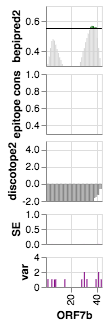

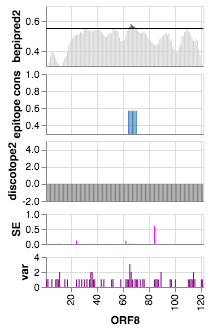

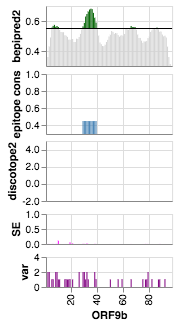

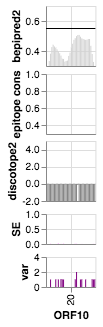

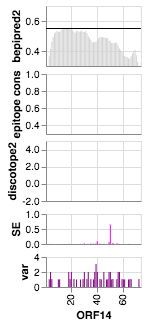

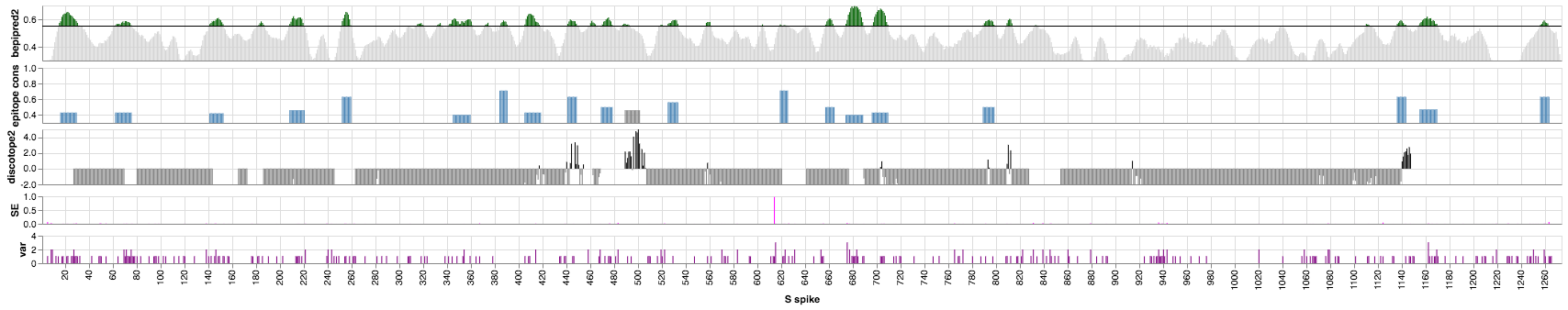


**Table S2**. Number of protein sequences from HCoV endemic strains found in the public databases. NCBI sequences originate from the Identical Protein Groups (IPG) database and UniProt sequences from the combined UniProtKB databases TrEMBL and Swiss-Prot with VARSPLIC extraction.

|  | **protein sequence database** | | |
| --- | --- | --- | --- |
| **HCoV strain** | **NCBI IPG** | **UniProtKB** | **ViPR** |
| HKU1 | 259 | 490 | 1,489 |
| 229E | 310 | 457 | 651 |
| OC43 | 1,033 | 1,591 | 5,648 |
| NL63 | 492 | 991 | 1,777 |
| Total | 2,094 | 3,529 | 9,565 |

**Table S3.** Structures and models used for the prediction of conformational epitopes. Min and max positions with structural information are relative to the proteins and may differ from residue numberings in PDB structures and models.

| **protein** | **structure** | **chain** | **min** | **max** | **source** | **method** | **reference** |
| --- | --- | --- | --- | --- | --- | --- | --- |
| M membrane protein | C1906TS131_2 | A | 1 | 222 | CASP | de-novo TrRosetta |  |
| N nucleoprotein | 6vyo | A | 50 | 173 | PDB | crystallography |  |
| N nucleoprotein | 6wji | A | 257 | 364 | PDB | crystallography |  |
| ORF3a | C1905TS405_5 | A | 1 | 275 | CASP | de-novo Kiharalab_Z |  |
| ORF6 | C1907TS073_2 | A | 1 | 61 | CASP | de-novo BISCORE |  |
| ORF7a | 6w37 | A | 16 | 81 | PDB | crystallography |  |
| ORF7b | C1910TS131_3 | A | 1 | 43 | CASP | de-novo TrRosetta |  |
| ORF8 | C1908TS131_1 | A | 1 | 121 | CASP | de-novo TrRosetta |  |
| ORF10 | C1909TS210_3 | A | 1 | 38 | CASP | de-novo Kiharalab |  |
| S spike | 6vxx | A | 27 | 1147 | PDB | cryo-EM | Walls 2020 |
| nsp1 | 15661 | A | 12 | 127 | Robetta | homology modelling |  |
| nsp2 | C1901TS156_1 | A | 2 | 639 | CASP | de-novo AlphaFold |  |
| nsp3 PL-PRO | 15659 | A | 1 | 110 | Robetta | homology modelling |  |
| nsp3 PL-PRO | 6vxs | A | 207 | 373 | PDB | crystallography |  |
| nsp3 PL-PRO | 15810 | A | 412 | 540 | Robetta | homology modelling |  |
| nsp3 PL-PRO | 15811 | A | 589 | 676 | Robetta | homology modelling |  |
| nsp3 PL-PRO | 6w9c | A | 748 | 1060 | PDB | crystallography |  |
| nsp3 PL-PRO | 15814 | A | 1091 | 1202 | Robetta | homology modelling |  |
| nsp3 PL-PRO | C1904TS401_1 | A | 1571 | 1927 | CASP | de-novo AlphaFold |  |
| nsp4 | C1902TS210_4 | A | 1 | 500 | CASP | de-novo Kiharalab |  |
| nsp5 3CL-PRO | 6yb7 | A | 1 | 306 | PDB | crystallography |  |
| nsp6 | C1903TS210_4 | A | 1 | 290 | CASP | de-novo Kiharalab |  |
| nsp7 | 7bv2 | C | 2 | 64 | PDB | cryo-EM | Yin 2020 |
| nsp8 | 7bv2 | B | 78 | 191 | PDB | cryo-EM | Yin 2020 |
| nsp9 | 6w4b | A | 5 | 113 | PDB | crystallography |  |
| nsp10 | 6w75 | B | 18 | 139 | PDB | crystallography |  |
| nsp12 RdRp | 7bv2 | A | 32 | 919 | PDB | cryo-EM | Yin 2020 |
| nsp13 helicase | 6jyt | A | 1 | 596 | PDB | crystallography | Jia 2020 |
| nsp14 ExoN-N7-MTase | 15671 | A | 1 | 527 | Robetta | homology modelling |  |
| nsp15 NendoU | 6vww | A | 92 | 346 | PDB | crystallography | Kim 2020 |
| nsp16 MTase | 6w75 | A | 2 | 298 | PDB | crystallography |  |

**Table S4**. Epitope scoring table. <excel spreadsheet>

**Table S5.** ELISA testing results summary (positive defined as values greater than the mean of negative controls added to 3-times the standard deviation of the mean).

| **Antigen** | **# SARS-CoV-2 samples** | | **# negative controls** | | **Sensitivity** | **Specificity** |
| --- | --- | --- | --- | --- | --- | --- |
|  | **tested** | **positive** | **tested** | **positive** |  |  |
| S-RBD | 78 | 72 | 22 | 0 | 92.3% | 100% |
| N-Nterm | 78 | 68 | 22 | 0 | 87.2% | 100% |
| Mix, S-RBD + N-Nterm | 80 | 75 | 106 | 3 | 93.8% | 97.2% |

**Table S6.** List of nucleoprotein sequences in the ViPR database from Seattle endemic HCoV strains matching the predicted dominant epitope nprot_10 on the Nucleoprotein dimerization domain. The SARS-CoV-2 epitope sequence and the matching endemic sequence is shown above the table.

nprot_10 KPRQKRTAT

match KPRQKRSPN

| **VIPR ID** | **Isolate** |
| --- | --- |
| APU51924 | Human coronavirus OC43 HCoV_OC43/Seattle/USA/SC831/2016 |
| APU51934 | Human coronavirus OC43 HCoV_OC43/Seattle/USA/SC622/2016 |
| APU51935 | Human coronavirus OC43 HCoV_OC43/Seattle/USA/SC9741/2016 |
| ARA15425 | Human coronavirus OC43 HCoV_OC43/Seattle/USA/SC2269/2016 |
| ARK08639 | Human coronavirus OC43 HCoV_OC43/Seattle/USA/SC2924/2015 |
| ARK08655 | Human coronavirus OC43 HCoV_OC43/Seattle/USA/SC2770/2015 |
| ARK08665 | Human coronavirus OC43 HCoV_OC43/Seattle/USA/SC2730/2015 |
| ARK08674 | Human coronavirus OC43 HCoV_OC43/Seattle/USA/SC2476/2015 |
| ARK08683 | Human coronavirus OC43 HCoV_OC43/Seattle/USA/SC2345/2015 |
| ARU07572 | Human coronavirus OC43 HCoV_OC43/Seattle/USA/SC2481/2015 |
| ARU07591 | Human coronavirus OC43 HCoV_OC43/Seattle/USA/SC2854/2015 |
| ARU07598 | Human coronavirus NL63 HCoV_NL63/Seattle/USA/SC2940/2015 |
| ARU07614 | Human coronavirus OC43 HCoV_OC43/Seattle/USA/SC3118/2015 |
| QEG03735 | Human coronavirus NL63 HCoV_NL63/Seattle/USA/SC0179/2018 |
| QEG03744 | Human coronavirus OC43 HCoV_OC43/Seattle/USA/SC0682/2019 |
| QEG03752 | Human coronavirus NL63 HCoV_NL63/Seattle/USA/SC0768/2019 |
| QEG03761 | Human coronavirus OC43 HCoV_OC43/Seattle/USA/SC0810/2019 |
| QEG03771 | Human coronavirus OC43 HCoV_OC43/Seattle/USA/SC0839/2019 |
| QEG03778 | Human coronavirus OC43 HCoV_OC43/Seattle/USA/SC0841/2019 |
| QEG03798 | Human coronavirus OC43 HCoV_OC43/Seattle/USA/SC9430/2018 |
| QEG03805 | Human coronavirus OC43 HCoV_OC43/Seattle/USA/SC9428/2018 |
| QEG03818 | Human coronavirus OC43 HCoV_OC43/Seattle/USA/SC0776/2019 |

**Table S7.** GISAID acknowledgement by authors and centers. <excel spreadsheet>
